# Methylmercury-induced cytotoxicity and oxidative biochemistry impairment in dental pulp stem cells: the first toxicological findings

**DOI:** 10.7717/peerj.11114

**Published:** 2021-06-10

**Authors:** Renata Duarte de Souza-Rodrigues, Bruna Puty, Laís Bonfim, Lygia Sega Nogueira, Priscila Cunha Nascimento, Leonardo Oliveira Bittencourt, Roberta Souza D’Almeida Couto, Carlos Augusto Galvão Barboza, Edivaldo Herculano Corrêa de Oliveira, Marcia Martins Marques, Rafael Rodrigues Lima

**Affiliations:** 1 Institute of Arts Sciences, Federal University of Pará (UFPA), Belém, Pará, Brazil, Brazil; 2 Laboratory of Functional and Structural Biology, Institute of Biological Sciences, Federal University of Pará (UFPA), Belém, Pará, Brazil, Brazil; 3 Laboratory of Tissue Culture and Cytogenetics, Environment Section, Evandro Chagas Institute, Ananindeua, Pará, Brazil; 4 Faculty of Dentistry, Federal University of Pará (UFPA), Belém, Pará, Brazil; 5 Department of Morphology, Federal University of Rio Grande do Norte, Natal, Rio Grande do Norte, Brazil; 6 Graduation Program, School of Dentistry, Ibirapuera University (UNIb), São Paulo, Brazil

**Keywords:** Methylmercury (MeHg), Dental pulp stem cells, Cell viability, Cell metabolism, Oxidative stress

## Abstract

**Background:**

Methylmercury (MeHg) is a potent toxicant able to harm human health, and its main route of contamination is associated with the consumption of contaminated fish and other seafood. Moreover, dental amalgams are also associated with mercury release on human saliva and may contribute to the accumulation of systemic mercury. In this way, the oral cavity seems to be the primary location of exposure during MeHg contaminated food ingestion and dental procedures but there is a lack of literature about its effects on dental tissues and the impact of this toxicity on human health. In this way, this study aimed to analyze the effects of different doses of MeHg on human dental pulp stem cells after short-term exposure.

**Methods:**

Dental pulp stem cells from human exfoliated deciduous teeth (SHED) were treated with 0.1, 2.5 and 5 µM of MeHg during 24 h. The MeHg effects were assessed by evaluating cell viability with Trypan blue exclusion assay. The metabolic viability was indirectly assessed by MTT reduction assay. In order to evaluate an indicative of antioxidant defense impairment, cells exposed to 0.1 and 5 µM MeHg were tested by measuring glutathione (GSH) level.

**Results:**

It was observed that cell viability decreased significantly after exposure to 2.5 and 5 µM of MeHg, but the metabolic viability only decreased significantly at 5 µM MeHg exposure, accompanied by a significant decrease in GSH levels. These results suggest that an acute exposure of MeHg in concentrations higher than 2.5 µM has cytotoxic effects and reduction of antioxidant capacity on dental pulp stem cells.

## Introduction

Mercury is a heavy metal, highly toxic to human health, that can occur naturally in the environment or by anthropogenic sources. It exists in three different forms: elemental (or metallic, Hg^0^), inorganic (mercuric chloride, Hg^2+^) and organic (ethyl-and methylmercury, MeHg) (Lima et al., 2018; WHO, 2007). Each of these forms has different levels of toxicity, various sources and routes of exposure. Organic mercury is considered as the most hazardous and, toxicologically, the most bioavailable form of mercury exposure (Abass et al., 2018; Budnik & Casteleyn, 2019). Sources and routes of mercury exposure include industrial process, resulting from human activity as mining; health care, from the use of thermometers and blood pressure monitors; traditional practices, as some therapies and religions; and food chain, from MeHg bioaccumulated in fish and seafood (WHO, 2007).

MeHg is the most frequent form of exposure after consumption of contaminated fish (Crespo-López et al., 2005). It has high liposolubility and can easily cross tissue membranes barrier primarily at oral cavity and then at gastrointestinal tract (Hong, Kim & Lee, 2012; Nogara et al., 2019). In addition, literature has also emphasized the relevance of Hg release from amalgam filling to saliva (Björkman, Sandborgh-Englund & Ekstrand, 1997; Zimmer et al., 2002; Yilmaz & Adisen, 2018; Pizzichini et al., 2002). The elemental mercury from amalgam restorations may be methylated by some bacteria (*Streptococcus minor*, *Streptococcus mutans* and *Streptococcus sanguis*) present in oral microbiome (Lyttle & Bowden, 1993; Jirau-Colón et al., 2019), becoming a source for local toxicity, ingestion and further systemic distribution of MeHg.

Once MeHg is absorbed, either by food consumption or amalgam fillings, it reaches different organs as brain, kidney, liver, and bones (Roos et al., 2010; Li, Feng & Qiu, 2010). However, only few studies have investigated the effects of MeHg on oral structures. Some of these focused on the amount of organic and inorganic mercury in human saliva (Leistevuo et al., 2001) and recently our group have shown the impact of mercury accumulation and toxicity on rat salivary glands (Lima et al., 2018; Farias-Junior et al., 2017; Bittencourt et al., 2017) and human periodontal ligament fibroblast (Nogueira et al., 2019). Nevertheless, no studies have been specifically evaluated the effect of MeHg on human dental pulp cells. Based on that, the understanding of different oral cells behaviour under MeHg exposure still necessary to fully comprehend MeHg-induced toxicity.

Dental pulp is a non-mineralized oral tissue composed of soft connective tissue, with vascular and nervous components that occupies the central pulp cavity. This dental tissue contains different types of cells: endothelial cells, neurons, fibroblasts, osteoblasts, osteoclasts, odontoblasts and stem cells (Nuti et al., 2016). Dental stem cells are multipotent, undifferentiated and highly proliferative (Miura et al., 2003), and they are recruited after dental pulp tissue injury when the first line of defense cells, the odontoblasts, are affected (Harada et al., 2008). These cells are easily isolated by a noninvasive procedure without ethical concerns (Yamada et al., 2019). Additionally, dental pulp stem cells have been studied in regenerative medicine in autologous stem cells therapies and allogenic tissue-engineering approaches showing relevant therapeutic competences in orofacial, cardiovascular, corneal, neurologic, hepatic, renal, diabetic, muscular dystrophy and auto-immune conditions, in both animal and human models, and in recent human clinical trials (Nakashima et al., 2017; Botelho et al., 2017; Ferrarotti et al., 2018).

In this perspective, the translational background of this study was the eventual dental pulp stem cells exposure to MeHg after systemic distribution of the toxicant by diet, (e.g.) by ingesting contaminated food and/or the mercury released from amalgam restoration, and further entrance on dental pulp by dentinal tubules. This exposure could have implications on the dental pulp homeostasis. Thus, the aim of the present study was to investigate the effects of different doses of MeHg on stem cells from human dental pulp after acute exposure. Our alternative hypothesis is that exposure to MeHg is capable of triggering oxidative and cytotoxic impairments on dental pulp stem cells. As a null hypothesis, there will be no differences in these parameters between cells exposed and not exposed to MeHg.

## Materials & methods

### Cell culture

Stem cells were isolated from a human exfoliated supernumerary deciduous tooth (35 region) of a 11 years-old girl. The tooth presented partial root resorption and no caries nor periodontal disease. The cells were nominated PDH1 lineage and were used in this study. These cells were donated by the Basic Research Laboratory of the School of Dentistry, University of São Paulo, São Paulo, Brazil. This study was approved by the Human Research Ethics Committee of the School of Dentistry, University of São Paulo, São Paulo, Brazil under Protocol #106/11CAAE 03511012.5.0000.075. Aliquots of cells between passages 8 and 12 kept stored in liquid nitrogen were thawed and diluted in Dulbecco’s Modified Eagle’s Medium (DMEM) supplemented with 10% fetal bovine serum (Gibco^TM^, Waltham, MA, USA), penicillin (10 U/ml), streptomycin (10 µg/ml) and fungizone (1%). The cells were maintained in an incubator at 37 °C in humid atmosphere containing 5% CO_2_. Cell growth was monitored daily under a phase contrast microscope, and the culture medium was changed every 2 days.

To ensure that the cells, kept the stem cell immunoprofile even after high passages, they were characterized again. For this, an aliquot of cells was evaluated by flow cytometry using the Human MSC Analysis Kit (BD Stemflow™, BD Biosciences, San Jose, CA, US), which permits to identify mesenchymal stem cells by detecting positive (CD105 PerCP-Cy5.5, CD73 APC, CD90 FITC) and negative (CD45, CD34, CD11b, CD19, HLA-DR PE) expression of cell surface markers.

### Experimental groups and exposure protocol to methylmercury

The experimental groups were as follows:

- Control: cells maintained into fresh culture medium (DMEM + 10% SBF);
- 0.1 µM MeHg: cells exposed to DMEM + 10% SBF + 0.1 µM MeHg;
- 2.5 µM MeHg: cells exposed to DMEM + 10% SBF + 2.5 µM MeHg;
- 5.0 µM MeHg: cells exposed to DMEM + 10% SBF + 5 µM MeHg.

MeHg (CH_3_HgCl, 115-09-3: Sigma-Aldrich, USA) stock solution was prepared in ultrapure water (H_2_0 u), filtered through a 0.22 μm membrane and kept at 4 °C. Right before experiments, serial dilutions were made in DMEM + 10% FBS to achieve 0.1, 2.5 and 5 µM MeHg.

In order to bring cell exposure closer to what occurs in human exposure, the lowest MeHg concentration chosen in our study is related to the Hg levels detected in chronically exposed riverine population in the Amazon river (Castilhos et al., 2015). The 2.5 and 5 µM MeHg were based on previous in vitro studies with oral cavity cells with the human periodontal ligament fibroblast (hPLF) (Nogueira et al., 2019). The 5 µM MeHg exerted significant toxic effects on viability and cell metabolism, while the 2.5 µM MeHg is within the range of concentration that significantly affected cell metabolism but minor changes on viability.

For MeHg exposure, PDH1 cells were seeded into 96-well plates (1 × 10^4^ cells per well). After 24 h incubation in a humid atmosphere containing 5% CO_2_ at 37 °C for attachment, the culture medium was replaced by medium plus MeHg, according to the experimental groups. Cells were exposed to MeHg for 24 h and then assessed by the following assays.

### Cell viability analysis

Trypan blue exclusion assay was used to assess cell viability. After 24 h of exposure to MeHg, the medium was removed, the cells were washed with Hank’s salt solution and then dissociated with trypsin/EDTA. Cells were centrifuged for 5 min at 5,000 rpm and resuspended in 30 µl of fresh culture medium. Ten microliters of cell solution were diluted in 10 µl of trypan blue and cells were counted and classified as non-viable (stained blue) and viable (unstained) in a Newbauer chamber. The percentage of viable cells was obtained by the following calculation:

}{}PLC\,\left(\% \right) = 100 \times \displaystyle{{viable\;cells} \over {total\;cells}}

### Metabolic viability

Cell metabolic viability was obtained by measuring the MTT reduction assay. After 24 h of exposure to MeHg, the medium was removed and it was added 100 µl MTT solution for 2 h in a humidified atmosphere containing 5% CO_2_ and 37 °C. After this time, the MTT solution was discarded and 100 µl of DMSO were added to elute the formazan crystals. Immediately after the end of the test procedures, the absorbance was read in a Glomax equipment (Promega, Madison, WI, USA) using a 570 nm filter. For data analysis, the absorbance results obtained were normalized by the number of viable cells as an indicative of cell metabolism, a new approach proposed by Rai et al. (2018).

### GSH levels measurement

**Figure 1:**
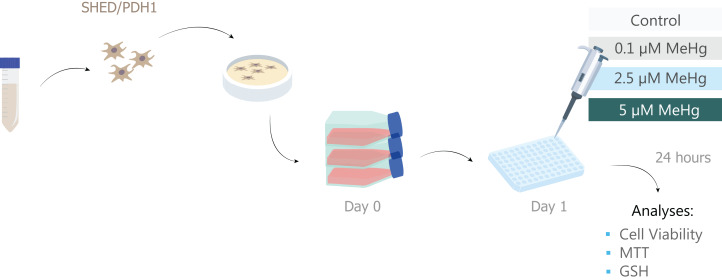
Sample description and experimental stages.

As indicative of oxidative stress, GSH levels were measured using GSH-Glo Kit (Promega, Madison, WI, USA) according to the manufacturer’s instructions. This test quantifies reduced glutathione based on the luciferase conversion reaction. For this test, only two MeHg concentrations were used: 0.1 and 5 µM MeHg, which represent non-toxic and toxic values, respectively, under the experimental conditions of the present study. After 24 h of exposure to MeHg, medium was removed and GSH-Glo reagent (luciferin-NT + glutathione S-transferase + GSH-Glo reaction buffer) was added to the samples and incubated for 30 min at room temperature. Thereafter, 100 µl of luciferin detection reagent were added. The samples were equilibrated at room temperature for 15 min and the luminescence signal was read using the Glomax multidetector system (Promega). The luminesce signal were used as an indicative of reduced glutathione on each well and was normalized by the number of viable cells. All experimental steps are summarized in Fig. 1.

### Statistical Analyses

The data were tabulated and results expressed as mean ± standard deviation (SD). Analyses were performed by one-way ANOVA and Tukey post-test, considering the level of significance *p* < 0.05. ANOVA assumptions (data normality through Shapiro–Wilk test) were previously verified. GraphPad Prism 7.0 software (GraphPad Software Inc., La Jolla, CA, USA) was used for all data analyses.

## Results

### PDH1 characterization

**Figure 2:**
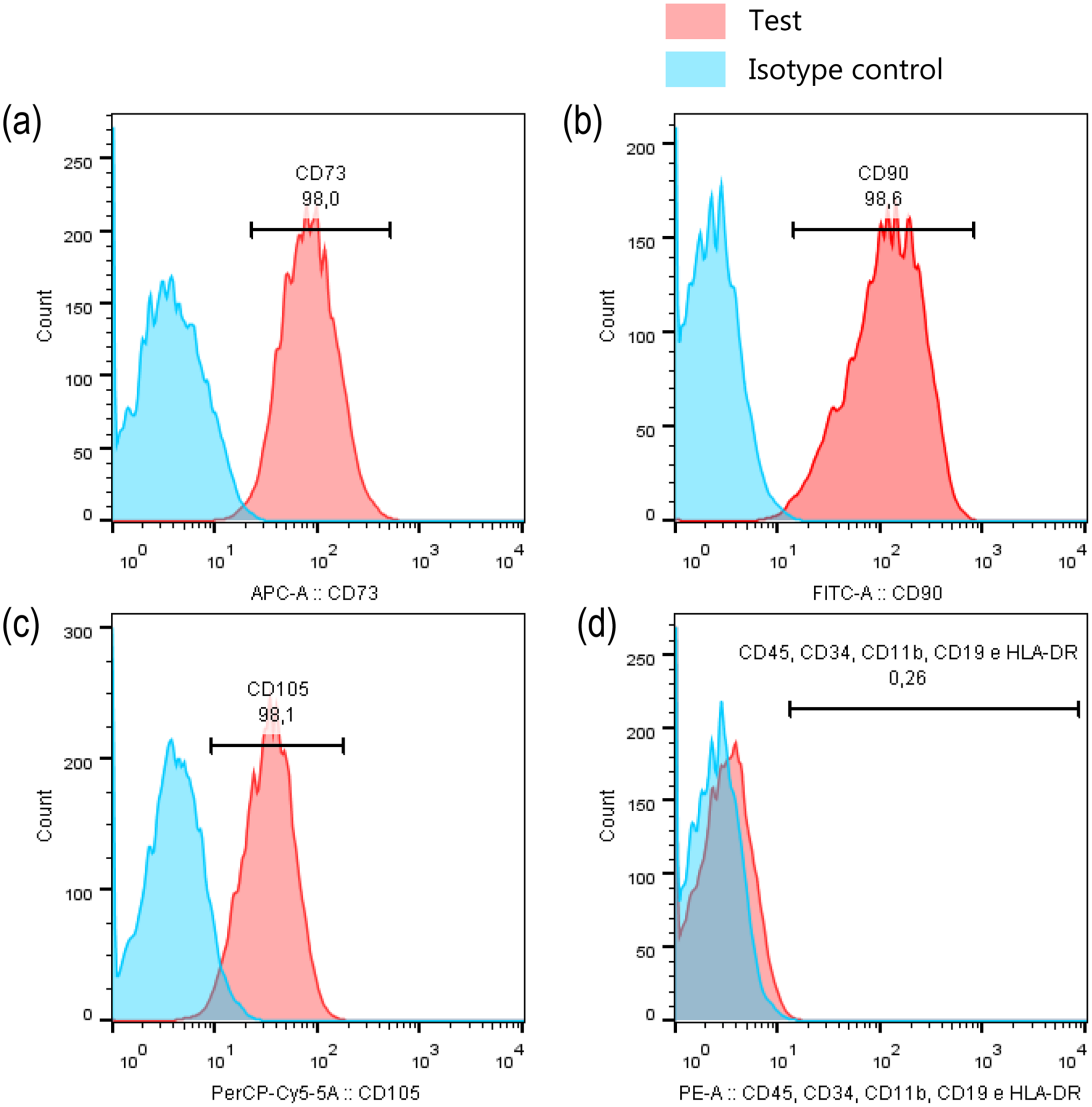
PDH1 characterization. Expression of stem cell markers in PDH1 cells: (A) CD90, (B) CD73, (C) CD105 and (D) negative cocktail.

Before MeHg experimental exposure procedures on dental pulp cells, we first performed the imunoprofile characterisation to ensure that cells were not changing their main characteristic of stem cells over passages. Our results showed that PDH1 cell were positive for the surface markers CD90 (98.0%, Fig. 2A), CD73 (98.6%, Fig. 2B) and CD105 (98.1%, Fig. 2C), and only 0.26% of cells were positive for CD45, CD34, CD11b, CD19, and HLA-DR, indicating their stem cell nature signature (Fig. 2D).

### MeHg exposure causes cell death in a concentration-dependent manner

**Figure 3:**
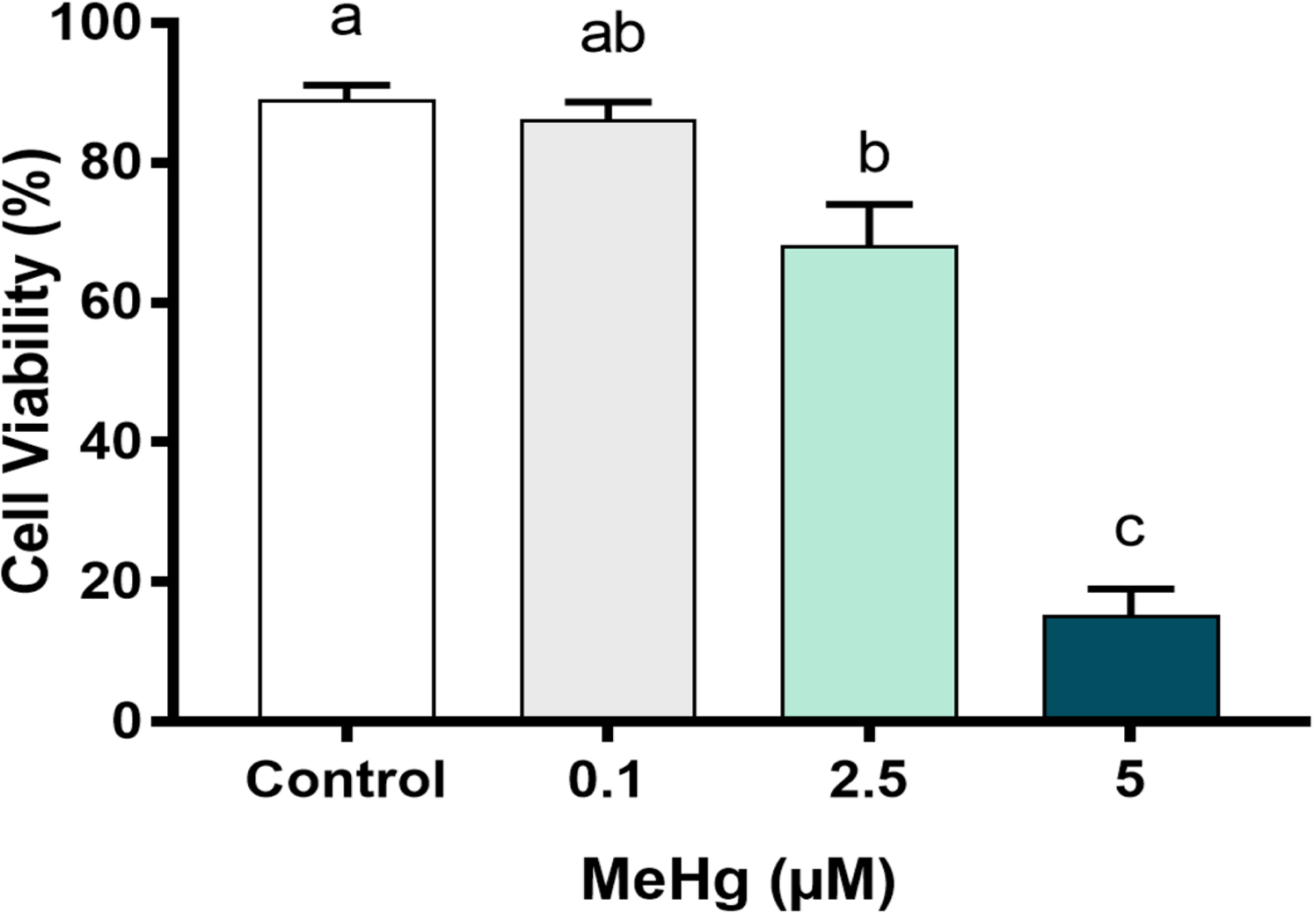
Effects of MeHg exposure for 24 h (in vitro) on dental pulp stem cells viability (PDH1 lineage). The data were compared by one-way ANOVA followed by the Tukey’s test (*p* < 0.05). Different overwritten letters show significant statistical difference.

To better understand the MeHg concentration-related toxicity on PDH1 cells, we first checked early damage events to cell viability and cell metabolism, after the exposure to three different concentrations that are usually found on Amazon riverside population blood plasma. After 24 h of MeHg exposure, cell viability decreased significantly in cells exposed to 2.5 µM MeHg and 5 µM MeHg. The highest cytotoxicity was observed at 5 µM MeHg (*p* < 0.01; F [3, 8] = 72.44, Fig. 3) while cell viability on 0.1 µM MeHg were similar to those of control group.

### High MeHg concentrations impair metabolic viability of dental pulp stem cells

**Figure 4:**
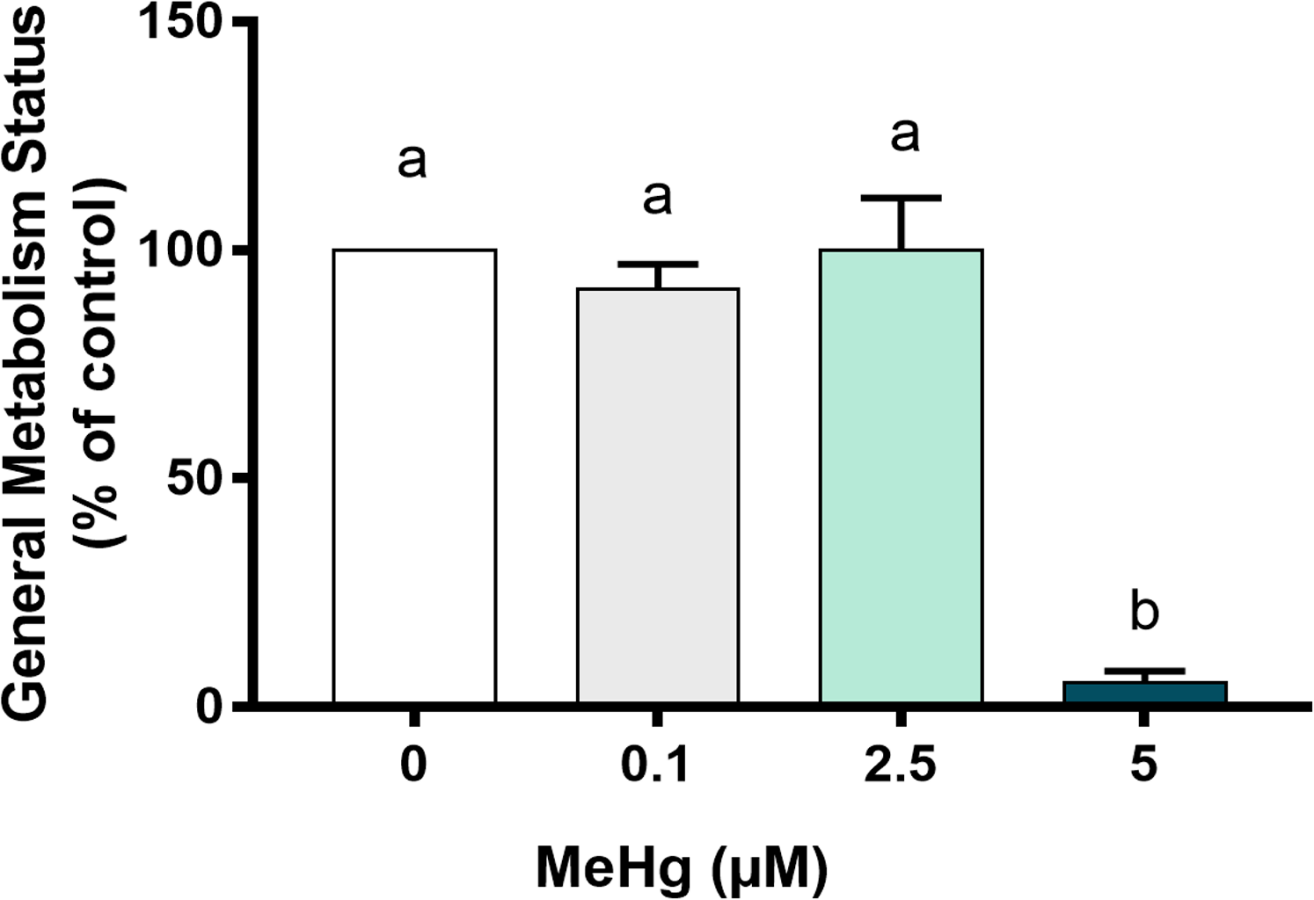
Effects of MeHg exposure for 24 h (in vitro) on cell general metabolism status, by MTT reduction method, of dental pulp stem cells (PDH1 lineage). The data were compared by one-way ANOVA followed by the Tukey’s test (*p* < 0.05). Different overwritten letters show significant statistical difference.

Knowing that cells with lower metabolic activity could be arrested to death over time, we next have evaluated how MeHg could impair cell metabolism. Our results showed that the metabolic viability after exposure to 5 µM MeHg was significantly decreased in comparison to all other groups (*p* < 0.01; F [3, 8] = 50.4). Exposure to 0.1 µM MeHg and 2.5 µM MeHg led to metabolism similar to the control group (Fig. 4).

### Oxidative biochemistry imbalance is increased by the higher MeHg concentrations

**Figure 5:**
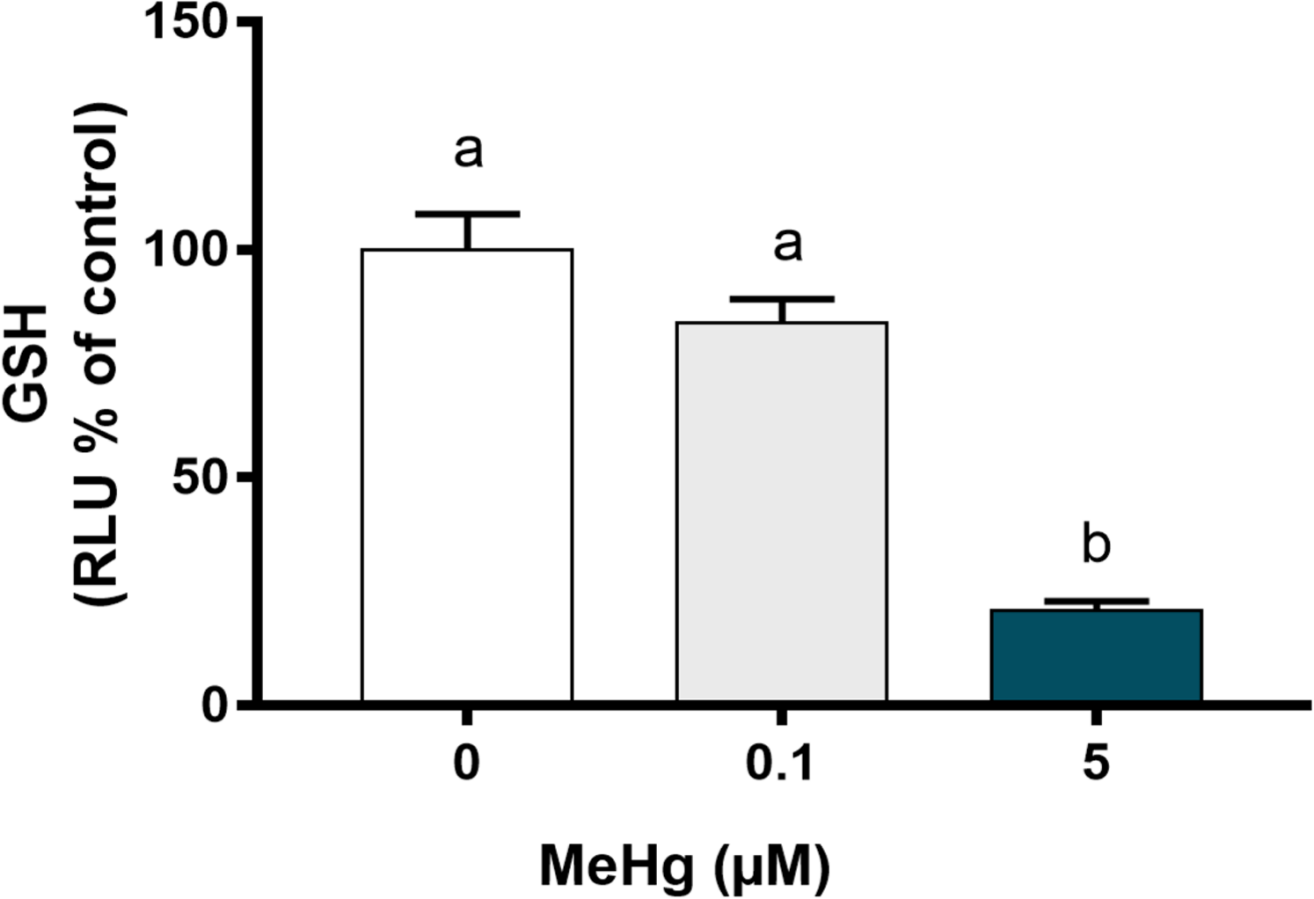
Effects of MeHg exposure for 24 h (in vitro) on total glutathione (GSH) levels of dental pulp stem cells (PDH1 lineage). The data were compared by one-way ANOVA followed by the Tukey’s test (*p* < 0.05). Different overwritten letters show significant statistical difference.

As MeHg-induced toxicity is related to oxidative stress, we further have analyzed whether PDH1 cells were able to activate defense mechanism to contain MeHg-induced damage. Reduced glutathione levels were evaluated as GSH are recognized as the primary defense of oxidative stress. Our results showed that GSH levels decreased significantly after exposure to 5.0 µM MeHg in comparison to the control group (*p* < 0.01; F [2, 6]) while no changes were observed on 0.1 µM MeHg (Fig. 5).

## Discussion

To the best of our knowledge, the present study is the first to show the damages triggered by in vitro MeHg exposure on dental pulp stem cells. Our results demonstrate that MeHg was able to promote a decrease in cell viability, affecting the cell metabolic viability and impairing cellular antioxidant defenses by reducing the levels of total GSH in concentrations considered toxic.

MeHg is one of the most toxic species of mercury to human health. The oral cavity structures and cells has recently appeared as target of mercury accumulation and toxicity. Although there is a statement that amalgam filling may contribute to systemic mercury toxicity (Lyttle & Bowden, 1993; Jirau-Colón et al., 2019) it is well established that the main form of mercury exposure occurs through the ingestion of contaminated fish and seafood. In this perspective, as mercury is a highly accumulative metal, people who eat fish contaminated with MeHg and have amalgams fillings are highly exposed to this toxicant (Jirau-Colón et al., 2019).

As mentioned before, in order to bring cell exposure closer to what occurs in human reality, the lowest MeHg concentration chosen in our study is related to the Hg levels detected on plasma samples of exposed riverine population in the Amazon river (Castilhos et al., 2015). In the present study, stem cells derived from human exfoliated supernumerary deciduous teeth (SHED; PDH1 lineage) were used. These cells were initially described by Miura et al. (2003), as a population of postnatal stem cells which possess an extensive proliferation rate and multipotent differentiation, meaning that, among the varied types of stem cells found in human body, it is one of the least differentiated. After tooth injuries as caries and dental procedures, when the first layer of cells, the odontoblast layer, is destroyed, these stem cells that are located more deeply in dental pulp, in sub odontoblastic layer, are activated, migrate to the injured area, and take place of the destroyed odontoblasts, being fundamental to tooth homeostasis and pulp repair (Harada et al., 2008). Thus, in this case, MeHg exposure would first reach these mesenchymal stem cells in dental pulp through the dentinal tubules, especially in the presence of residual or secondary caries lesions.

In stem cell research, an important point is to characterize the cells before the experiments are performed. In the present study, PDH1 cells were validated as mesenchymal stem cells (MSC) based on the immunoprofile of stem cell specific markers. Data analysis indicated that most (≥98%) cells positively expressed CD73 (ecto-5′-nucleotidase), CD90 (Thy1), and CD105 (endoglin) while only 0.26% of the cells expressed CD45, CD34, CD11b, CD19, and HLA-DR surface molecules. These findings are consistent with the criteria to define human MSC established by the International Society for Cellular Therapy (Dominici et al., 2006).

Our group has shown several damages caused by mercurial exposure in experimental models evaluating salivary glands and central nervous system (Lima et al., 2018; Bittencourt et al., 2019; Bittencourt et al., 2017; Corrêa et al., 2020; Freire et al., 2019; Santana et al., 2019; Teixeira et al., 2019), which are often associated with the mercury systemic distribution, that increases its levels in different organs. Moreover, despite the fact that Minamata disease is the most prominent outcome in patients with mercurial intoxication (Harada, 1995), several other disorders have also been reported in patients living in regions of mercurial exposure (Puty et al., 2019). In this perspective, the systemic distribution of the metal may play the major role to deliver mercury to dental pulp.

For the assessment of cell viability and overall metabolism status, different MeHg concentrations that ranged from 0.1 to 5 µM were tested. As expected, MeHg cytoxicity increased in a dose-dependent manner whereas the number of viable cells decreased significantly at 2.5 μM and 5 μM of MeHg, and the cell metabolic viability decreased significantly only at 5 µM. The slight decrease on cell viability with no changes on cell metabolism at 2.5 µM of MeHg seems to be related to the induction of cell survival responses by the activation of protective signaling pathways and consequently high metabolic demand at low concentrations of MeHg (Levonen et al., 2004). Similar results for cell viability were found by Nogueira et al. (2019) in a study using human periodontal ligament fibroblasts (hPLF) cells and different MeHg concentrations. But in the referred study, the metabolic viability decreased with a lower dose of MeHg, 2 µM. It is important to note that in the present study, MTT assay was used considering a new approach as proposed by Rai et al. (2018), as an indicator for metabolic viability evaluation and not in the way it is usually used, i.e., an appropriate indicator of mitochondrial function or directly related to the number of living cells (Green & Leeuwenburgh, 2002). A recent study (Huang et al., 2019) investigated the effects of environmental chemicals, such as MeHg, paraquat and bisphenol A, on neural stem cells originated from human cord blood (HUCB-NSCs) and, although the authors used a different experimental model, as well as different approaches, they showed that 50 and 100 nmol/L MeHg and the other chemicals, when tested separately, significantly reduced the proliferation cellular only in 72 h, demonstrating the sensitivity of these cells to smaller amounts of MeHg in exposure times higher than that used by us. Another study (Wang et al., 2016), that also used different experimental model and approaches, investigated the effects of various doses of MeHg (0, 10 and 50 nM) on human embryonic neural progenitor cells (hNPCs) for 24 h, and only 50 nM MeHg showed significant differences to the control group in term of cell viability, which shows that hNPCs were sensitive to lower MeHg doses in the same exposure time that we used.

The intracellular mercury is often reported to trigger changes in the homeostasis of human systems by the oxidative stress mechanism (Syversen & Kaur, 2006). Among the pathways of oxidative imbalance, the ability of MeHg to interact with groups of proteins and non-protein molecules, such as the glutathione (GSH), is identified as one of the main ways (Yin et al., 2007). Alterations in GHS levels play a crucial role in MeHg-induced toxicity, mainly to the nervous system (Farina, Rocha & Aschner, 2011) and also to cell lines obtained from oral cavity, as recently proven (Nogueira et al., 2019).

The depletion of total GSH only occurred in PDH1 cells exposed to 5 µM. GSH participates of many critical cellular processes, but one of most important function is related to the antioxidant system, acting as a reducing agent for reactive oxygen species (ROS) (Antunes Dos Santos et al., 2018). Then, the results would be related to interactions between MeHg and thiols groups, which can be found in low mass molecules, as glutathione. This interaction occurs because MeHg has a relative high affinity for electrons, that causes its specifically high reactivity with nucleophilic centers, as sulfhydryl (-SH or thiol) groups, forming stable excretable GS-MeHg complexes (Farina & Aschner, 2019). The reduction of total glutathione level (GSH) is an endpoint of the imbalance between the production of reactive oxygen species and antioxidant defenses, but the oxidative stress is only confirmed by the presence of excessive levels of reactive oxygen species and/or damage to macromolecules, DNA, proteins and lipids (Antunes Dos Santos et al., 2018). In this way, our results may suggest that on PDH1 cells MeHg may induce oxidative stress mediated by the reduction on antioxidant capacity mediated by glutathione, but additional studies that evaluate changes on the macromolecules cited before are necessary to confirm that.

## Conclusions

Under the experimental conditions of this study, the results demonstrate that an acute exposure to MeHg impairs the cell viability, metabolic viability and oxidative biochemistry of dental pulp stem cells. Further studies with longer exposure times and other biochemical analyses are required to elucidate how these changes could affect the dental pulp metabolism and homeostasis.

## Supplemental Information

10.7717/peerj.11114/supp-1 Supplemental Information 1 Raw data.

10.7717/peerj.11114/supp-2 Supplemental Information 2 Effects of exposure of MeHg for 24 h (in vitro) on cell viability, cell general metabolism status and total glutathione (GSH) levels of dental pulp stem cell (PDH1 lineage).

## Additional Information and Declarations

### Data Availability

The following information was supplied regarding data availability: Raw data is available in the Supplemental Files.
