# Supplementary material for: Methylmercury-induced cytotoxicity and oxidative biochemistry impairment in dental pulp stem cells: the first toxicological findings
Source: PeerJ. 2021 Jun 10;9:e11114. doi: 10.7717/peerj.11114 (PMC8199917; doi:10.7717/peerj.11114)
Supplement: Supplemental Information 1 [file peerj-09-11114-s001.pdf]

	Group A	Group B	Group C	Group D
	0	0.1	2.5	5
	Y	Y	Y	Y
1	83.53	78.05	76.17	20.60
2	90.40	89.51	60.31	39.59
3	92.49	87.22	68.24	12.36

	Group A	Group B	Group C	Group D
	0	0.1	2.5	5
	Y	Y	Y	Y
1	100	104.34	116.147	0.482
2	100	81.45	83.660	2.970
3	100	96.83	99.900	5.520

	Group A	Group B	Group C
	0	0.1µM	5µM
	Y	Y	Y
1	100.0	8447.00	2078.00
2	978.0	8792.00	2803.00
3	954.0	8098.00	1409.00
